# H3.3K27M Mutation Controls Cell Growth and Resistance to Therapies in Pediatric Glioma Cell Lines

**DOI:** 10.3390/cancers13215551

**Published:** 2021-11-05

**Authors:** Andria Rakotomalala, Quentin Bailleul, Clara Savary, Mélanie Arcicasa, Maud Hamadou, Paul Huchedé, Audrey Hochart, Audrey Restouin, Remy Castellano, Yves Collette, Emma Dieny, Audrey Vincent, Pierre-Olivier Angrand, Xuefen Le Bourhis, Pierre Leblond, Alessandro Furlan, Marie Castets, Eddy Pasquier, Samuel Meignan

**Affiliations:** 1 Tumorigenesis and Resistance to Treatment Unit, Centre Oscar Lambret, F-59000 Lille, France; andria.rakotomalala.etu@univ-lille.fr (A.R.); quentin.bailleul@roquette.com (Q.B.); m-arcicasa@o-lambret.fr (M.A.); a-furlan@o-lambret.fr (A.F.); 2 University of Lille, CNRS, Inserm, CHU Lille, UMR9020-U1277-CANTHER—Cancer Heterogeneity Plasticity and Resistance to Therapies, F-59000 Lille, France; audrey.vincent@inserm.fr (A.V.); pierre-olivier.angrand@univ-lille1.fr (P.-O.A.); xuefen.le-bourhis@univ-lille.fr (X.L.B.); 3 Lyon Cancer Research Center, Inserm U1052, 69008 Lyon, France; Clara.SAVARY@lyon.unicancer.fr (C.S.); maud.hamadou@etu.univ-lyon1.fr (M.H.); Paul.HUCHEDE@lyon.unicancer.fr (P.H.); emma.dieny@free.fr (E.D.); pierre.leblond@ihope.fr (P.L.); marie.castets@lyon.unicancer.fr (M.C.); 4 CHU Lille, Hematology and Transfusion, F-59000 Lille, France; audrey.hochart@chru-lille.fr; 5 Centre de Recherche en Cancérologie de Marseille, Aix-Marseille Université, Inserm, CNRS, Institut Paoli Calmettes, 13009 Marseille, France; Audrey.Restouin@inserm.fr (A.R.); Remy.Castellano@inserm.fr (R.C.); Yves.collette@inserm.fr (Y.C.); eddy.pasquier@inserm.fr (E.P.); 6 Lyon Pediatric Hematology and Oncology Institute, 69008 Lyon, France

**Keywords:** H3.3K27M, glioma, child, resistance to therapies, radiotherapy

## Abstract

**Simple Summary:**

Although the involvement of the H3.3K27M mutation in Diffuse Midline Glioma tumorigenesis is now established, its role in their resistance to treatments and, therefore, in their fatal outcome remains poorly documented. Here, thanks to our models of H3.3K27M induction in pediatric glioma cells, we finally shed light on this crucial issue. Hence, we demonstrate here for the first time that H3.3K27M can increase cell radioresistance capabilities independently of TP53 alterations. Moreover, thanks to a drug library screening, we evidenced that this mutation can, depending on the cellular context, drastically modulate the response of these cells to different classes of compounds, thus paving the way for new therapeutic strategies. Altogether, our results provide here the proof that, beyond its role in tumorigenesis, the presence of H3.3K27M mutation by itself alters the response to treatments of pediatric glioma cells.

**Abstract:**

High-grade gliomas represent the most lethal class of pediatric tumors, and their resistance to both radio- and chemotherapy is associated with a poor prognosis. Recurrent mutations affecting histone genes drive the tumorigenesis of some pediatric high-grade gliomas, and H3K27M mutations are notably characteristic of a subtype of gliomas called DMG (Diffuse Midline Gliomas). This dominant negative mutation impairs H3K27 trimethylation, leading to profound epigenetic modifications of genes expression. Even though this mutation was described as a driver event in tumorigenesis, its role in tumor cell resistance to treatments has not been deciphered so far. To tackle this issue, we expressed the H3.3K27M mutated histone in three initially H3K27-unmutated pediatric glioma cell lines, Res259, SF188, and KNS42. First, we validated these new H3.3K27M-expressing models at the molecular level and showed that K27M expression is associated with pleiotropic effects on the transcriptomic signature, largely dependent on cell context. We observed that the mutation triggered an increase in cell growth in Res259 and SF188 cells, associated with higher clonogenic capacities. Interestingly, we evidenced that the mutation confers an increased resistance to ionizing radiations in Res259 and KNS42 cells. Moreover, we showed that H3.3K27M mutation impacts the sensitivity of Res259 cells to specific drugs among a library of 80 anticancerous compounds. Altogether, these data highlight that, beyond its tumorigenic role, H3.3K27M mutation is strongly involved in pediatric glioma cells’ resistance to therapies, likely through transcriptomic reprogramming.

## 1. Introduction

Tumors of the central nervous system (CNS) are the most frequent solid tumors in children, representing around 25% of pediatric cancers [1]. Among those, glial tumors, or gliomas, represent approximately half of the contingent [2]. They are subdivided into low-grade glioma (LGG, WHO grade I and II) and high-grade glioma (HGG, WHO grade III and IV), which strongly differ in their prognosis, with a 5-year survival of 91% for LGG and only 46% for HGG patients [3]. Among HGG, the Diffuse Intrinsic Pontine Glioma (DIPG), which accounts for 15 to 20% of childhood CNS tumors [4], constitutes a unique entity. Indeed, DIPGs have a dismal prognosis with a median survival of less than 12 months and a 2-year survival rate of around 10% [5]. Surgical resection is precluded by the location in the brainstem and by their highly infiltrative properties.

DIPGs are almost exclusively pediatric and to date, the extrapolation of data obtained from adult glioma patients has proven insufficient to increase the patient’s survival [6]. They are extremely resistant to all current therapies, including chemotherapies, targeted therapies and ionizing radiations. Numerous clinical trials have been conducted for 40 years, but none significantly increased patient survival [7]. The current standard of care remains fractionated radiotherapy (1.8 Gy daily for 30 days) [8], which is only palliative.

The discovery of the recurrent H3K27M somatic mutations in histone H3 genes provided new insights into the biology of DIPG [9,10,11,12]. These mono-allelic mutations lead to the replacement of a lysine by a methionine in position 27 of the histone H3 proteins. They occur more frequently in the *H3F3A* gene (around 73% of cases), coding the H3.3 variant, but are also found in the *HIST1H3B/C* gene, coding for the H3.1 variant (around 26% of cases). Mutation in the H3.2 variant was also detected in a few tumors (<1% of cases). H3K27M mutations have a dominant-negative effect that results in a global loss of the H3K27me3 epigenetic marks in the genome [13]. They were shown to be an early event and to drive tumorigenesis, in combination with *TP53* mutation and *PDGFRA* amplification [14,15,16]. Accordingly, the 2016 update of the WHO classification introduced a new entity, the Diffuse Midline Glioma H3K27M mutant [17].

Of note, epigenetic alterations are common in pediatric HGG. Indeed, most H3K27 non-mutated DIPG and PFA (Posterior Fossa A) ependymomas display a similar epigenetic rewiring as Diffuse Midline Glioma H3K27M mutant, notably driven by EZHIP over-expression [18,19,20,21,22]. Furthermore, H3G34R/V mutations are observed in supratentorial pediatric HGGs, and cause other epigenetic alterations responsible for aberrant transcriptional programs in these cancers [9,12,23,24]. Altogether, these data highlight the crucial role of epigenetic alterations for pHGG development. Owing to this context, therapies targeting epigenetic alterations linked to the H3K27M mutations have been evaluated in DIPG cells [25]. For example, promising pre-clinical results were obtained using GSK-J4, an inhibitor of the demethylase JMJD3, which is responsible for the demethylation of H3K27 [26]. However, it seems that this drug is rapidly transformed in vivo in GSK-J1, which has a limited therapeutic potential due to decreased cell permeability [26,27]. In addition, panobinostat, an HDAC inhibitor, was highlighted in a screening of pharmacological molecules against DIPG cells, independently of the H3K27M status [28,29]. However, the ability of this drug to improve the survival of DIPG-xenografted mice was only partial [28,30]. Similarly, the role of the H3.3K27M mutation in the regulation of the Notch pathway led to the evaluation of its inhibition in DIPG models, without a clear demonstration of the efficacy of this approach so far [24,31].

Beyond these targets, to the best of our knowledge, the specific impact of H3K27M mutation in cell resistance to a wide range of therapies has not been elucidated. In that frame, we here evaluate the impact of inducing H3.3K27M mutation in both low- and high-grade pediatric glioma cells. We introduced the K27M-mutated *H3F3A* gene in pLGG and pHGG cell lines and assessed the consequences on epigenetic marks, gene expression, and biological processes, including cell growth and clonogenicity. Finally, we evaluated, for the first time, the specific impact of the mutation on the response to a wide panel of anticancer drugs but also to ionizing radiations, which remain the standard of care treatment in the clinic.

## 2. Materials and Methods

### 2.1. Cell Culture

Human pediatric high-grade glioma cell lines SF188 and KNS42 (grade IV, glioblastoma multiform) and low-grade glioma cell line Res259 (grade II, diffuse astrocytoma) were kindly provided by Dr Chris Jones (The Institute of Cancer Research, Sutton, UK). Among other genetic alterations, SF188 cells harbor *MYC* and *CCND1* amplifications, KNS42 cells have a low-level copy number gain of *PI3KCA* locus and, Res259 cells harbor an amplification of *PDGFRA* and a deletion of *CDKN2A/B*. A more detailed phenotypic and molecular characterization of these cell lines was previously described by Bax and colleagues [32]. All cell lines were grown as a monolayer in DMEM medium supplemented with 10% fetal bovine serum (FBS), L-glutamine, non-essential amino acid solution and antibiotics cocktail (Life Technologies, Carlsbad, CA, USA). All cell lines were free of mycoplasma contamination. Cells were incubated in a humid atmosphere at 37 °C with 5% CO_2_.

### 2.2. Molecular Cell Engineering

Cells were dissociated, collected and 1 × 10^6^ cells was transfected using Cell Line NucleofectorTM Kit V (Lonza, Bâle, Switzerland) with 1µg of plasmid containing K27M mutated *H3F3A* gene fused with the *mCherry* gene, and bearing a resistance gene for Hygromycin B. As a control, cells were transfected with a similar plasmid containing the WT *H3F3A* gene. After 2 days, Hygromycin B was added at 400 µg/mL. After 2 weeks of selection, cells were sorted with an ARIA III SORP (Becton-Dickinson, Franklin Lakes, NJ, USA) flow cytometer using the 561 nm laser line and the emission filter 610/20.

### 2.3. Confocal Microscopy

Cells were seeded in a chambered 8-well coverslip µ-Slide (Ibidi, Gräfelfing, Germany) at a density of 25,000 cells per well. After 24 to 48 h of incubation, cells were labeled with Hoechst (NucBlue, Invitrogen, Waltham, MA, USA) to visualize the cell nuclei. Cells were then observed by confocal laser scanning microscopy (LSM 880, Zeiss, Oberkochen, Germany), using a Laser diode (405 nm) and a Laser line (561 nm), and the respective appropriate emission filters (445/50 and 605/70) with a 20× objective.

### 2.4. Immunoblotting and Histone Marks Levels Analysis

For western blot analysis, histones were collected using a histone extraction kit (Abcam, Cambridge, UK). Protein concentration was determined by BCA Assay (Bio-Rad, Hercules, CA, USA). Membranes were saturated with a solution of bovine serum albumine 5% and washed with TBS-Tween 0.2% solution after antibody incubations. The following primary antibodies were used: Rabbit anti-H3K27M (Abcam ab190631, Cambridge, UK), rabbit anti-H3.3 (Abcam ab97968, Cambridge, UK), rabbit anti-H4 (Cell Signaling 13919, Danvers, MA, USA), rabbit anti-H3K27me1/me2/me3 (Cell Signaling 84932/9728/9733, Danvers, MA, USA), rabbit anti-H3K27ac (Abcam ab195417, Cambridge, UK). All primary antibodies were diluted at ^1^/_1000_. The secondary anti-rabbit antibody coupled with HRP (Cell Signaling, Danvers, MA, USA) was diluted at ^1^/_2000_ in BSA buffer. Signals were revealed using Luminata Crescendo Western HRP Substrate (Merck Millipore, Burlington, MA, USA) and detected with the Fusion Solo S system (Vilber Lourmat, Marne-la-Vallée, France).

Complementarily, to quantify H3K27me1/2/3 and H3K27ac marks, a multiplexed screening of histone H3 post-translational marks was also performed using the Luminex xMAP technology assay through an ActiveMotif service delivery. Briefly, 750,000 cells were harvested, centrifuged, and pellet samples were sent to ActiveMotif. Relative histone H3 concentrations in the samples were determined using the H3 Total beads. Multiplex assays were performed according to relative abundance using sample volumes normalized for histone H3 concentration. The assay plate was read on the Luminex LX200 Instrument. Samples were tested in duplicate read in a Magpix instrument and data exported as CSV files. Data sets with equivalent H3 Total signals were selected for downstream analysis. Net median fluorescent Intensity (Net MFI) associated with each PTM-specific bead was expressed as a ratio relative to Histone H3 Total signals for each well. Ratio values were averaged for each sample input amount, and percent change in the ratio relative to the reference samples was determined. T-tests at the 95% confidence interval were performed to assess PTM changes. A control lysate was also included to check the multiplex assay execution.

### 2.5. Growth Assay

Cells were seeded in 96-well plates (250 cells/well for SF188 and Res259 and 500 cells/well for KNS42), and the plate was placed in an Incucyte video microscope (Essen Bioscience, Royston, UK) that measured the cell confluency daily throughout the experiment.

### 2.6. Cell Cycle Analysis

Cells were collected and incubated 30 min with Vybrant™ DyeCycle™ Violet Stain (ThermoFischer Scientific, Waltham, MA, USA). Cell cycle was analyzed by flow cytometry using CytoFLEX (Beckman Coulter, Brea, CA, USA), using a violet laser at 430 nm, with emission filter 450/45. Analysis was realized using Modfit software.

### 2.7. RNA-Seq Analysis

RNA was extracted using the Nucleospin RNA kit following the manufacturer’s instructions (Macherey-Nagel, Hoerdt, France) with on-column DNA digestion. RNA quality was evaluated using the 2100 Bioanalyzer Instrument (Agilent, Santa Clara, CA, USA). RIN scores above 9 were considered acceptable for RNA-seq experiments. RNA libraries were prepared using the TruSeq™ RNA Library Preparation Kit v2 and run on the Illumina NovaSeq 6000 Sequencing System (NovaSeq 6000 SP Reagent Kit, 200 cycles, Illumina, San Diego, CA, USA) to generate 2 × 100 million paired end reads in the iGenSeq Platform of the Paris Brain Institute.

Quality control of human pediatric glioma cell lines SF188, KNS42 and Res259 RNA-seq data was assessed using default parameters of FastQC (version 0.11.9; https://github.com/s-andrews/FastQC; visited on 13 March 2021). Pseudo-alignment based on the human transcriptome annotation Ensembl v96 and transcript-level quantification were performed using Kallisto (version 0.46.1; https://github.com/pachterlab/kallisto; visited on 13 March 2021) [33] with “bias“ and “plaintext” parameters. Transcript-level expression was summarized into gene-level expression using tximport R library (version 1.18.0; https://github.com/mikelove/tximport; visited on 13 March 2021) [3] using “type = kallisto”, “ignoreTxVersion = True” and “countsFromAbundance = lengthScaledTPM” parameters. The Ensembl v96 annotation used by tximport was provided by the AnnotationHub R library (version 2.22.1; https://github.com/Bioconductor/AnnotationHub; visited on 13 March 2021) [34]. Finally, Transcripts Per Kilobase Million (TPM) expression values were then transformed in log^2^(TPM + 2).

Pathway RespOnsive GENes (PROGENy) is a computational method and R software package that infers pathway activity from gene expression data [35]. This approach was built by analyzing large-scale gene expression changes from short-term perturbation experiments to capture the primary response to different stimuli. Z-scores were calculated from gene expression changes for a large compendium of publicly available perturbation experiments that yield a definition of gene expression signatures specific to 11 pathways EGFR, MAPK, PI3K, VEGF, JAK- STAT, TGFb, TNFa, NFkB, Hypoxia, p53-mediated DNA damage response and Trail (apoptosis). We used the progeny function (progeny R library v.1.14.0; https://github.com/saezlab/progeny; visited on 13 March 2021) on the full gene expression to quantify pathway activation for each cell line. Functional enrichment analyses were undertaken using the integrative web-based application EnrichR (https://maayanlab.cloud/Enrichr; visited on 13 March 2021).

### 2.8. Zebrafish Embryos Xenografts

For the local dissemination study, AB/TU zebrafish (*Danio rerio*) embryos were raised in a dedicated platform (Animalerie Zebrafish Rockefeller, Université de Lyon). Prior to injection, 2 × 10^6^ Res259-H3.3 and Res259-H3.3K27M cells were harvested, rinsed in serum-free medium and resuspended in 30 μL of PBS. At 48 h post-fecundation, zebrafish embryos were dechorionated with pronase (10165921001, Sigma-Aldrich, Saint-Louis, MO, USA), anesthetized with tricaine (E10521, Sigma-Aldrich, Saint-Louis, MO, USA) and injected with approximately 20 nL of either Res259-H3.3 or Res259-H3.3K27M cells in the yolk sac. Embryos were further incubated at 28 °C for 48 h in E3 medium. To quantify cells that disseminated locally in the yolk sac, embryos were fixed for 6 h in 4% paraformaldehyde (PFA) at 4 °C. Embryos were then embedded in 1% agarose columns and placed so that the yolk sac faces the lens for Selective Plane Illumination Microscopy (SPIM) imaging using a Zeiss LIGHTSHEET Z.1 with a W Plan-Apochromat 20×/1.0 detection objective (Zeiss, Oberkochen, Germany). Acquisitions were realized using the Zen 2014 SP1 black edition software and 3D reconstructions were made from all z-stacks using Arivis Vision4D 3.4 (Arivis AG, Rostock, Germany). A tumor was qualified as locally disseminated when more than five cells detached from the principal mass were detected.

### 2.9. Clonogenicity Assay and Ionizing Radiations

Cells were seeded into 12-well culture plates (200 cells/well). Six hours later, cells were irradiated with fractions of 0, 1, 2 or 3 Grays per day for 3 consecutive days using a linear accelerator XSTRAHL 100 (photons of 50 kV, dose rate of 1.25 Gy/min). After irradiation, cells were maintained for 10 days, then colonies were revealed by crystal violet staining. Colonies are defined to consist of at least 50 cells (approximately corresponding to the minimal colony size detectable by eye) and were manually counted using a colony counter pen [36]. The Plating Efficiency (PE) is the ratio (number of colonies counted/number of seeded cells) at 0 Gy and the Survival Fraction (SF) is the ratio (number of colonies after irradiation/(PE × number of seeded cells)). The results were represented as a survival curve (SF depending on the irradiation dose) on a logarithmic scale.

### 2.10. Screening of Chemotherapeutic Drugs

Drug sensitivity and resistance profiling was performed on human Res259, SF188 and KNS42 cell lines. The drug screening library included 80 substances consisting of a broad range of conventional chemotherapeutics and targeted agents (Table S1). Each compound was tested in four concentrations separated by one log at a constant DMSO level (1%). This allows covering four concentration logs and determining IC50 values. Briefly, 1250 living cells were seeded per well in 96-well plates and incubated in the presence of compounds in a humidified environment at 37 °C and 5% CO_2_. As described by Bax et al., PLOS One, 2009, low-grade (i.e., grade II) pediatric glioma Res259 cells displayed a shorter doubling time (approximately 24 h) compared to high grade (i.e., grade IV) glioma SF188 and KNS42 cells (doubling times of 26 h and 48 h, respectively). Therefore, we performed our drug sensitivity assays for 72 h according to the standards [32]. Cell viability was measured using the CellTiter-Glo luminescent assay (Promega, Madison, WI, USA) and the data were normalized to negative control wells (DMSO only). Half-maximal inhibitory concentration values (IC_50_) were deduced from dose-response curves obtained using GraphPad Prism software.

Subsequently, precise IC_50_ evaluation of the top 4 agents showing resistance in the K27M transfected cells was performed with the same protocol, this time with a range of 8 concentrations with a 2-fold increment between each concentration.

### 2.11. Statistical Analysis

All statistical analyses and fittings were performed with GraphPad PRISM software. Two-way ANOVA test was systematically applied to evaluate the significance of in vitro assays. Concerning experiments in zebrafish embryos, the Chi-square (χ^2^) test was used to assess the differences in the frequency of disseminated tumors compared to the total number of tumors in each group.

## 3. Results

### 3.1. Expression of the H3.3K27M Mutation Decreases H3K27 Trimethylation and Triggers Pleiotropic Transcriptomic Changes in Pediatric Glioma Cell Lines

To investigate the impact of the H3.3K27M mutation on pediatric glioma resistance to therapies, we established stable cell lines expressing the *H3F3A* mutated gene fused with the coding sequence for mCherry fluorescent protein in three pediatric gliomas cell lines: Res259 (WHO Grade II), SF188 and KNS42 (both WHO Grade IV). We thus obtained cells expressing an ectopic mutant histone H3.3, that we will refer to as H3.3K27M thereafter. As a control, we introduced a similar construct leading to the expression of the wild-type *H3F3A* gene in cells, thereafter referred to as H3.3.

**Figure 1:**
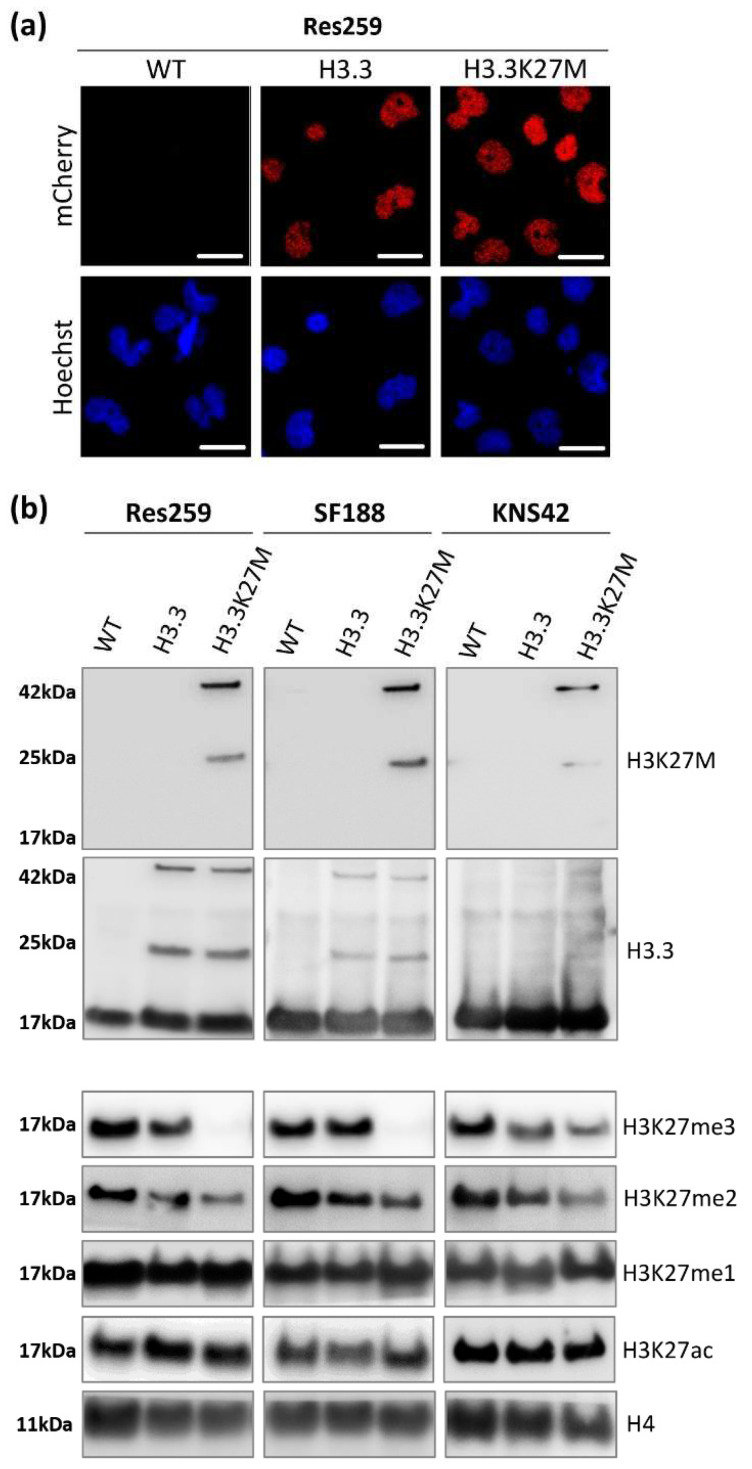
Validation and epigenetic characterization of the H3.3K27M-engineered cell models. (**a**). Microscopy pictures of the Res259 cell line, WT cells or cells expressing the H3F3A mutated (H3.3K27M) or unmutated (H3.3) gene. Fluorescence was acquired for the mCherry signal (top lane) or Hoechst signal (bottom lane). Scale bars 10 µm. (**b**). Western blot of H3K27M and H3.3 was performed on all cell lines. Other epigenetic marks were also evaluated as H3K27me3/me2/me1 and H3K27ac. H4 was used as a loading control.

We validated the nuclear location of our fusion proteins by fluorescence microscopy (Figure 1a and Figure S1A). Over 99% of cells were mCherry-positive as assessed by flow cytometry after amplification (Figure S1B), confirming the stable expression of our transgenes in our glioma cell models.

We next investigated the expression of H3.3K27M or its wild-type counterpart in our cell lines by western blot (uncropped version available in Figure S2). Thanks to an antibody specifically recognizing the K27M mutation, we confirmed the expression of the mutated protein, fused with mCherry at 42 kDa (25 kDa for the mCherry protein + 17 kDa for H3.3 protein) in the three different H3.3K27M cell lines (Figure 1b). Since no band was detected at the size of the endogenous H3.3 protein using this K27M specific antibody, we validated the absence of this mutation in the glioma cell lines that we selected for this study. Western blot against mCherry displayed the same pattern (Figure S1C), and so did the western blot against pan H3.3 (Figure 1b), with the addition of the bands corresponding to the endogenous wild-type H3.3 proteins. Interestingly, the introduction of our constructs triggered a similar expression level of exogenous H3.3 in H3.3K27M and H3.3 control cells for each cell line (Figure 1b), which warrants the goodness of our models to compare the importance of the H3.3K27M mutated protein with respect to its wild-type counterpart. Moreover, we could check that the level of H3.3 endogenous protein was not modified by our approach and that we did not trigger an aberrant overexpression of exogenous proteins (Figure 1b).

Since H3.3K27M mutation drives epigenetic alterations in patients, we further evaluated the level of relevant epigenetic marks in our cell models. We first checked the level of H3K27 tri- (me3), di- (me2) and mono- (me1) methylation by western blot. In all cell lines, the H3.3K27M mutation induced a strong decrease in the level of repressive marks, especially at the H3K27me3 level but also at the H3K27me2 one (Figure 1b). We confirmed the impact of our constructs on the H3K27me3 level using the Luminex^®^ technology to quantify histone post-translational modifications (Active Motif service delivery, Figure S1D). In contrast, both H3K27me1 and H3K27ac levels were not altered by the mutation in the three cell lines (Figure 1b).

**Figure 2:**
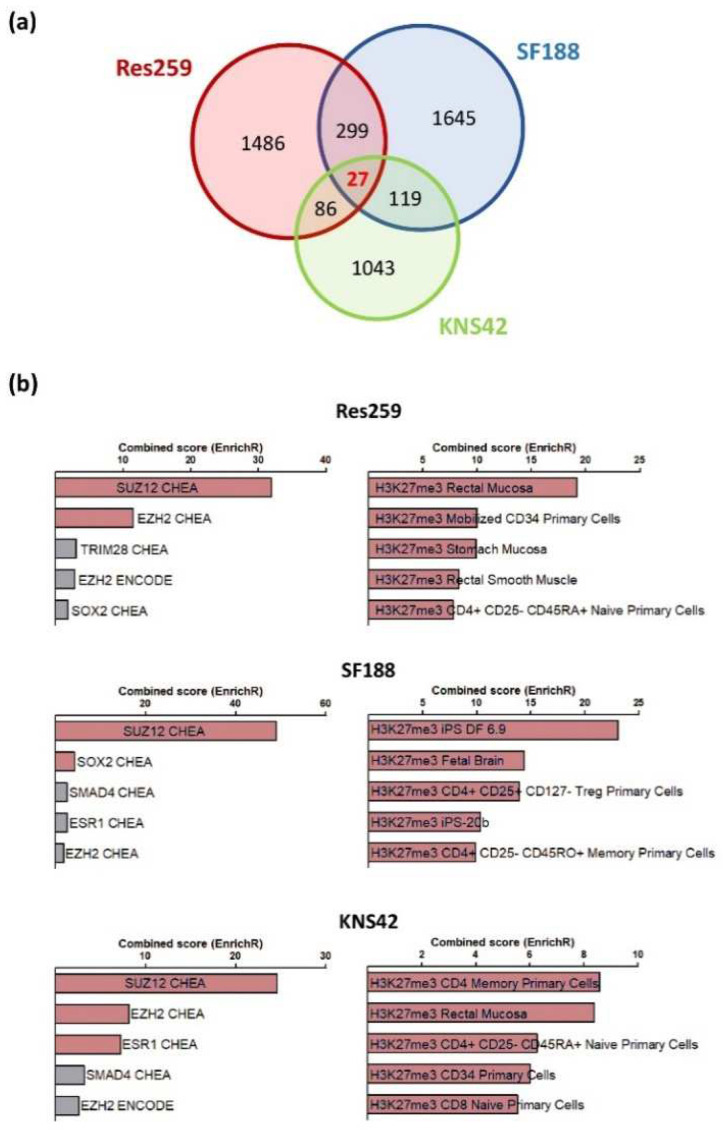
Transcriptomic reprogramming of K27M-expressing glioma cells. (**a**). Venn diagram representation of genes whose expression was changed by more than 2-folds in each H3.3K27M vs. control cell line. (**b**). Bar-graphs obtained via an EnrichR analysis, showing that genes whose expression is changed in each cell line correspond significantly to H3K27me binding regions, defined by Chip-seq analyses in the framework of the ENCODE and ChEA Consensus TFs from ChIP-X (left panel) and Epigenomics Roadmap HM ChIP-seq (right panel) (red bar show significant *p*-value).

To obtain a full insight into the molecular repercussions of the H3.3K27M mutation and of the associated epigenetic alterations, we performed a bulk RNAseq analysis to compare the transcriptome of Res259, KNS42 and SF188 cells expressing wild-type or mutant forms of the H3.3 histone. In all cell lines, we observed a wide panel of changes in gene expression patterns, with 1825, 2017, and 1275 genes whose expression was increased or decreased by more than two folds in mutant versus control cell lines in Res259, SF188, and KNS42 cells, respectively. However, only 27 genes were common to all cell lines, without enrichment in a particular signaling pathway (Figure 2a, Table S2). We could not determine whether these genes were differentially expressed in DIPG tumors due to the absence of publicly-available datasets for this rare form of childhood cancer.

Using the EnrichR database, we observed that genes whose expression was changed in each cell line correspond preferentially to an enrichment in signatures associated with H3K27 trimethylation and PRC2 (Polycomb Repressor Complex), when comparing H3.3K27M vs. control cells in the three cell lines (Figure 2b).

Collectively, these results indicate that the stable introduction of the H3.3K27M mutation in glioma cell lines is sufficient to reproduce the epigenetic changes observed in H3K27M mutated gliomas, with subsequent transcriptomic reprogramming that seems to depend on the cellular context.

We then analyzed in silico the activation of distinct pathways using the PROGENy algorithm, a bio-analysis tool developed to infer the activation of specific signaling pathways. We thus observed that the expression of the K27M led to alterations of pathways involved in the regulation of cell proliferation, notably a PI3K-Akt pathway activation in Res259 and SF188 mutant lines as compared to their wild-type counterparts (Figure S3).

### 3.2. H3.3K27M Increases Cell Growth and Clonogenic Properties of Res259 and SF188 Glioma Cells

**Figure 3:**
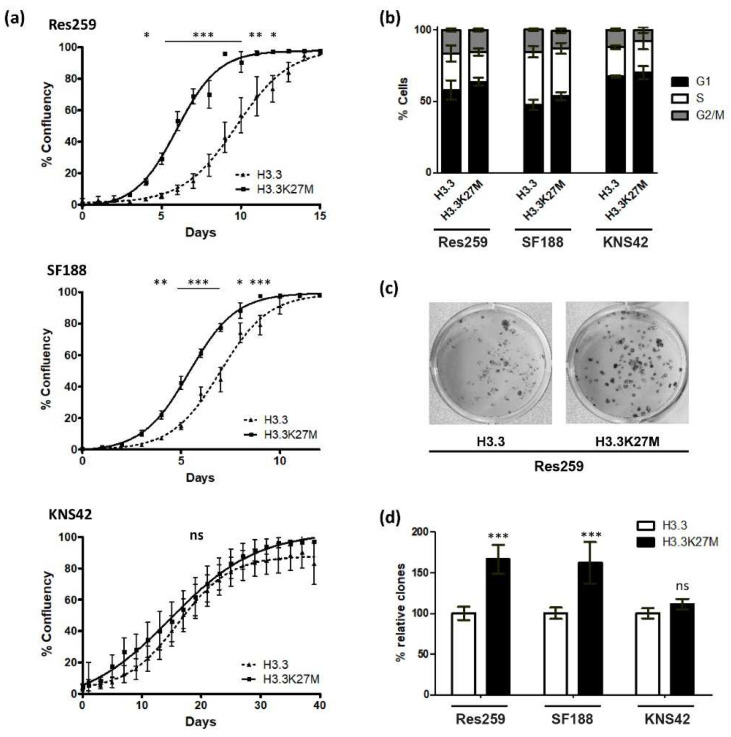
Effects of H3.3K27M induction on cell growth and clonogenic properties. (**a**) Confluency was measured every day using Incucyte. Cell growth curves give the % of confluency as a function of time in days. The assay was conducted in sextuplicate and repeated 3 times. Error bars represent ± SD of sextuplicates. Statistical significance was calculated with an Anova test (*** *p* < 0.001; ** *p* < 0.01; * *p* < 0.05; ns—non-significant) (dark line and squares: H3.3K27M; dotted dark line and triangles: H3.3). (**b**) Cell cycle distribution measured by flow cytometry using Vybrant™ DyeCycle™ Violet Stain. (**c**) Pictures of colonies formed by Res259-H3.3 and -H3.3K27M cells 10 days after seeding at low density. (**d**) Percentage of formed colonies normalized to H3.3 condition in each cell line. The experiment was realized in quadruplicate and repeated 3 times. Error bars represents ± SD of quadruplicates. Significance was evaluated using an Anova test. (*** *p* < 0.001, ns—non-significant).

We then decided to focus on the impact of the H3.3K27M mutation on glioma cell oncogenic properties. We first monitored cell growth by time-lapse microscopy and showed that Res259 and SF188 but not KNS42 H3.3K27M cells reached confluency before H3.3 control ones (Figure 3a). To further investigate these differences in cell growth, we evaluated cell cycle repartition by flow cytometry. We could not detect any significant differences between H3.3 and H3.3K27M cells for any of the three cell lines, even if we noticed heterogeneity in cell cycle repartition among them (Figure 3b and Figure S4A). These results were consistent with the cell growth curves that displayed similar slopes, even for Res259 and SF188 cells: in both cell lines, the difference between H3.3 and H3.3K27M cells rather relies on an initial delay for H3.3 cells to reach exponential growth.

To analyze the clonogenic potential of our cell lines, we then seeded them at low density in 12-well plates and quantified the proportion of cells that could form colonies. This number was much higher for Res259- and SF188-H3.3K27M cells when compared to control cells (66% and 62% increases for Res259 and SF188, respectively) (Figure 3c,d and Figure S4B). In contrast, and in agreement with their cell growth curves, KNS42 cells did not show significant differences in their ability to form colonies whether they expressed WT H3.3 or H3.3K27M (Figure 3d).

Taken together, these results suggest that H3.3K27M can modify the oncogenic properties of glioma cells, likely due to transcriptomic changes induced by the expression of the mutation.

Given the diffuse nature of DIPG, we then wondered whether H3.3K27M mutation could also impact their in vivo migratory properties. We used a zebrafish xenograft model to evaluate the local dissemination of control and H3.3K27M Res259 cells (Figure S5A,B). We observed a trend suggesting a higher propensity of H3.3K27M mutant cells to locally disseminate, as compared to control cells (52% vs. 27% of disseminating tumors), although this result did not reach statistical significance (Figure S5C).

### 3.3. H3.3K27M Promotes Resistance to Fractioned Radiotherapy in Res259 and KNS42 Glioma Cells

For more than 40 years, radiotherapy has remained the reference treatment for DIPG, even if these tumors eventually become refractory to this therapy. Thanks to our models, we could investigate whether the H3.3K27M mutation plays a role in this radioresistance.

**Figure 4:**
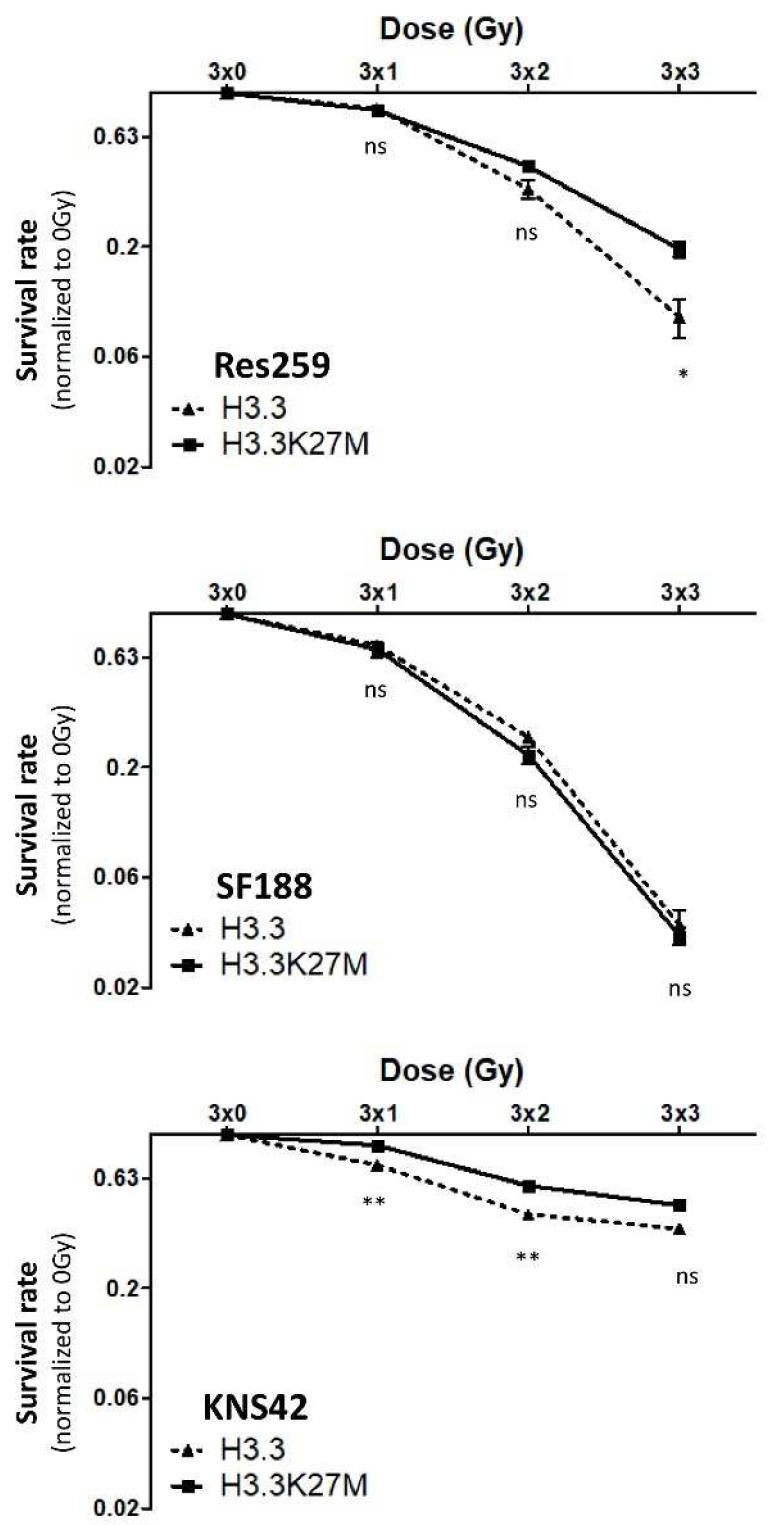
Effect of H3.3K27M induction on cell response to irradiations. The impact of H3.3K27M on cell resistance to ionizing radiations was determined by clonogenic assay. Cells were irradiated with fractions of 0, 1, 2 or 3 Grays per day for 3 consecutive days. After 10 days, colonies were counted. The survival rate (or fraction) is defined as the following ratio: (number of colonies after irradiation/(Plating Efficiency × number of seeded cells)). Results are representative of 3 independent experiments. ** *p* < 0.01; * *p* < 0.05; ns—non-significant.

To address this question, we applied a fractionated protocol of ionizing radiations (0, 1, 2 or 3 Gy) to our cell models every day for 3 consecutive days. After calculating the plating efficiency of each cell line, we were able to quantify the survival rate for each dose of irradiation. No significant difference in the radiosensitivity was observed between H3.3 and H3.3K27M cells in the high-grade glioma SF188 cell line, which displayed an exacerbated basal answer to ionizing radiations, with 80% decrease in survival rate already at 3 × 2 Gy (Figure 4, central panel). On the contrary, we showed that both H3.3K27M low-grade glioma Res259 and high-grade KNS42 cells were significantly more resistant to irradiation when compared to H3.3 cells (Figure 4, top and bottom panels).

These results thus show that H3.3K27M mutation can drive cell resistance to radiotherapy both in low-grade glioma and in high-grade glioma cell lines, which already present a partial radioresistance.

### 3.4. H3.3K27M Strongly Affects Drug Sensitivity in Low-Grade Glioma Cells

We finally evaluated the impact of the mutation on the Drug Sensitivity/Resistance Profile (DSRP) of glioma cells. A panel of 80 pharmacological agents, including conventional chemotherapeutic drugs and targeted therapies, were tested in our glioma models. The IC50 ratio was determined for each drug in H3.3K27M versus H3.3 cell line pairs. We set the cut-off to >2.5 and <0.4, to conclude on a chemo-resistant or chemo-sensitive effect of the mutation, respectively.

**Figure 5:**
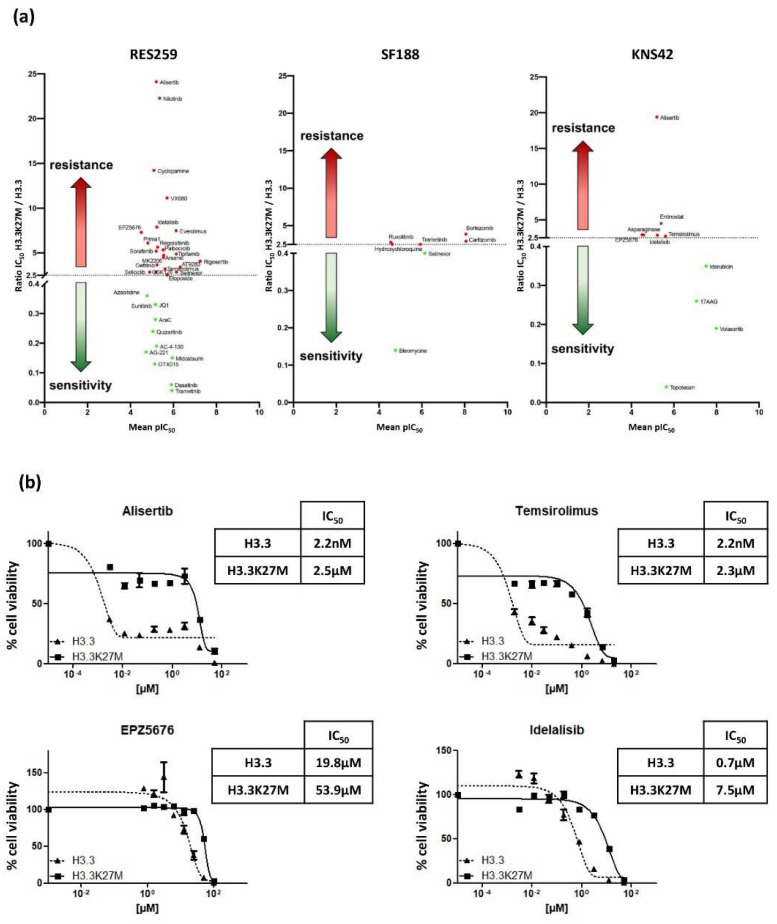
Impact of H3.3K27M on pediatric glioma cell response to a library of antitumoral drugs. (**a**) A library of 80 compounds was tested, each one at 4 different concentrations. Cells were seeded and incubated with compounds for 72 h. The ratio of H3.3K27M/H3.3 IC50 was calculated and reported. Red dots represent a ratio > 2.5 between H3.3K27M and H3.3, i.e., chemoresistance linked to the mutation. Green dots represent a ratio < 0.4, reflecting a chemo-sensitivity conferred by the mutation. (**b**) Res 259 cells were incubated and drugs were added 24 h after incubation. % of viability, relative to the condition 1% DMSO defined as 100% (control), was measured by CellTiter-Glo luminescent assay, 72 h after treatment. The IC_50_ values indicated in the tables were determined by fitting the survival curves. (H3.3 dotted dark line; H3.3K27M dark line).

The mutation differentially impacted the DSRP in the three cell lines (Figure 5a). It had very little impact on the drug sensitivity of SF188 and KNS42, with seven and nine drugs showing differential sensitivity, respectively. In sharp contrast, the mutation had a strong impact on the chemo-sensitivity of the Res259 cell line. Indeed, more than 40% of the tested drugs showed differential sensitivity in this cell line, with 22 drugs displaying increased IC_50_ (ratio ranging from 2.5 to 24.1) and 11 drugs displaying decreased IC_50_ (ratio ranging from 0.36 to 0.04). This suggests a global change in DSRP in this cell model.

It is noteworthy that, even if the mutation increases the chemoresistance of KNS42 cell for only six drugs, four of them exhibit a similar trend in the Res259 cells: Temsirolimus, which is an inhibitor of mTOR (IC_50_ ratios of 3.2 and 2.7 for KNS42 and Res259 cell lines, respectively); EPZ5676, that inhibits DOT1L methyltransferase (IC_50_ ratios of 7.3 and 2.9, respectively); Idelalisib, which is an inhibitor of phosphoinositide 3-kinase (PI3K) (IC_50_ ratios of 7.9 and 2.9, respectively), and Alisertib, which is an Aurora A kinase inhibitor (IC_50_ ratios of 24.1 and 19.4, respectively).

We further validated these results by refining the range of concentrations and confirmed the differences between Res259-H3.3 cells and Res259-H3.3K27M cells (Figure 5b).

Collectively, these results show that, beyond its impact on pediatric glioma cell radioresistance, H3.3K27M mutation also alters their DSRP.

## 4. Discussion

Although the H3.3K27M mutation was evidenced in pHGG years ago, and its impact on the epigenetic landscape has been well studied, little is known about its role in resistance to therapies. In order to address this issue, we designed pediatric glioma cell lines, stably expressing the H3.3 protein K27M-mutated or not.

The introduction of the mutation did not impact the endogenous H3.3 protein level, and the transgene expression level appeared similar between H3.3 and H3.3K27M conditions. We confirmed the relevance of our cell models by showing that the introduction of the H3.3K27M protein acted in a dominant manner on the endogenous H3.3 to drive the expected epigenetic modifications, such as the previously described global loss of H3K27me3 [13,15] and the decrease in H3K27me2 [37]. The impact of the H3K27M mutation on H3 acetylation is still controversial, with some studies reporting an increase in this acetylation mark in H3.3K27M DIPG cells [38,39], whereas others failed to identify significant changes [40,41]. Here, we did not observe any increase in the H3K27ac mark in our glioma background, which highlights the difficulty of drawing a generalist model on this mark.

Thanks to this relevant model generated in three different pediatric glioma cell lines, we were able to investigate the biological consequences of the H3.3K27M mutation. We first showed that the H3.3K27M mutation accelerates cell growth in Res259 and SF188. Although it was suggested from a murine DIPG model that H3.3K27M induced differences in cell cycle repartition, with an increase in the proportion of cells in S phase [42], we could not detect such differences in our models. From the appearance of growth curves, we suggest that H3.3K27M mutation could increase the propensity of cells to grow at low confluency, thereby increasing their proliferation rate in the first phases of our tests. This could explain why other studies, in which seeding concentrations were much higher, did not observe differences in cell growth of H3K27M mutated vs. unmutated cells [24,43]. Consistently, when we seeded cells at a very low density to measure their clonogenic potential, H3.3K27M cells produced a greater number of colonies when compared to control ones. This result also suggests that the K27M mutation could have a positive impact on stemness properties. Along this line, Silveira et al. showed that the inhibition of *H3F3A* expression by shRNA induced cell differentiation, concluding on the role of the mutation to maintain a stem-cell profile [44]. These observations were reinforced by Chen et al., who highlighted the activation of Notch pathway, involved in stemness, in DIPG H3.3K27M cellular models [24].

Of note, we did not observe such differences in proliferation/clonogenic properties between mutant vs. control KNS42 cells, suggesting that the impact of the mutation strongly depends on the cellular context, in line with its varied effect on the transcriptome observed in the different cell lines. Indeed, we observed that, although the introduction of the mutation was sufficient to induce a clear transcriptomic reprogramming of all three cell lines, only 0.5% of genes whose expression was altered by the mutation were common to all three models. Along this line, the increase in proliferative capacity, observed only in Res259 and SF188 cells, may be related to the activation of the PI3K-AKT pathway, evidenced with the PROGENy algorithm. The prediction of the oncogenic impact of the H3.3K27M mutation and the definition of associated therapeutic targets will thus have to take into account the impact of the cross-talk with the epigenetic environment and the cellular context in which it occurs.

In that frame, we then wondered whether the H3.3K27M mutation affected the response of pediatric glioma cells to therapies. Again, we observed a diversity of impact depending on the cellular context in which the H3.3K27M mutation is expressed. Indeed, we showed that Res259 and KNS42, but not SF188, H3.3K27M cells had a higher resistance to ionizing radiations in a fractionated schedule. Interestingly, the higher basal sensitivity to irradiation observed in SF188 mutant cells may suggest that the mutation reinforces radioresistance mechanisms already present in glioma cells such as Res259 and KNS42. In DIPG, resistance to radiotherapy was mostly suggested to result from the highly frequent *TP53* mutations [45]. In this study, since mutant and control Res259 and KNS42 differ only by the presence of the H3.3K27M mutation, our results clearly demonstrate for the first time that this! aberration is also sufficient to drive resistance to radiotherapy, in both low- and high-grade contexts.

Radiotherapy still remains the only standard of care for DIPG. Nevertheless, taking into account its partial and transient efficacy, fighting DIPG chemoresistance represents another challenge that our community must face. In 2015, Grasso and colleagues screened a library of anticancer drugs against 14 patient-derived DIPG cell cultures (one H3K27wt, four H3.1K27M-mutated, and nine H3.3K27M-mutated) and highlighted the efficiency of Panobinostat [28]. However, because of the presence of only one H3K27wt cell culture in their panel, authors were not able to evaluate the role of the mutation in chemoresistance. Our models allowed us to investigate this issue and suggest that the response to panobinostat is not altered by the mutation. Here, for the first time, we evaluated the specific impact of the H3.3K27M mutation on cell sensitivity/resistance to a representative panel of anticancer drugs. We showed that the mutation significantly altered the resistance to therapies mostly in a low-grade glioma context, with minimal impact in high-grade glioma cells. This suggests that, because of their own molecular alterations, H3.3K27M expression in high-grade glioma cells is less critical than in a low-grade one corresponding to a less aggressive disease. Of note, we report induced resistance to four common drugs in Res259- and KNS42-H3.3K27M cells, among which are idelalisib (PI3K inhibitor) and temsirolimus (mTOR inhibitor) that both target the PI3K-Akt/mTOR pathway. This result may seem contradictory with the extrapolation of the transcriptomic signature using the PROGENy algorithm, which suggests that the mutation H3.3K27M rather leads to the activation of the PI3K-AKT pathway. However, although still largely unknown, several mechanisms of resistance to this pathway have been described. Compensation for AKT inactivation induced by PI3K inhibitors was shown to result from an increase in IL-6 signaling [46], which could be interesting since IL-6 and IL-6R are, respectively, increased by more than six- and three-fold in Res259- and KNS42-H3.3K27M cells compared to control ones.

Furthermore, we also evidenced that H3.3K27M mutation conferred resistance to alisertib and EPZ5676 (also known as pinometostat), which inhibit Aurora kinase and DOT1L methyltransferase (the enzyme responsible for H3K79 methylation marks deposition), respectively.

The resistance to EPZ5676 seems particularly interesting since it suggests that H3K27M-induced alterations may cross-talk with other epigenetic marks so far not investigated in DIPG, such as H3K79me. Thus, it represents a possible target to combine with other Achille’s heels of mutated DIPG cells. In this sense, future studies will be aimed at unraveling the H3K79me amount and distribution along the genome in our models in order to better understand the H3.3K27M-mediated resistance to the DOT1L inhibitor EPZ5676, and propose novel therapeutic strategies based on the highlighted mechanisms.

This study was mainly carried out with in vitro models, which allowed us to combine several approaches and to obtain an integrated deeper insight into the role of H3.3K27M mutation in cell physiology and resistance to many therapies.

The processes evidenced in this work, and especially the efficacy of therapies, now require to be validated with in vivo models, notably patient-derived xenografts, in order to fully meet the translational aim of the study. This will be the topic of future investigations.

## 5. Conclusions

Although further analyses are required, these results highlight the fact that the H3.3K27M mutation can trigger mechanisms of drug/radiation resistance. Elucidation of the cross-talk between epigenetic/cellular contexts will thus be crucial to find the Achilles’ heel of tumor cells among the transcriptional alterations induced by the mutation and to devise more effective therapeutic combinations in the management of DMG.

## Data Availability

The data presented in this study are available on request from the corresponding author.

## Supplementary Materials

The following are available online at https://www.mdpi.com/article/10.3390/cancers13215551/s1, Figure S1: Validation of the H3.3K27M induction models, Figure S2: Uncropped Western blot, Figure S3: Pathway activation score ratio between K27M and H3.3 conditions in Res259, SF188 and KNS42 glioma cell lines, Figure S4: Impact of the H3.3K27M on cell cycle and clonogenic properties, Figure S5: Local dissemination of Res259-H3.3 and -H3.3K27M xenografts in zebrafish embryos, Table S1: Drug screening list, Table S2: Common genes differentially expressed between H3.3 and H3.3K27M in the three cell lines.
