# Supplementary material for: H3.3K27M Mutation Controls Cell Growth and Resistance to Therapies in Pediatric Glioma Cell Lines
Source: Cancers (Basel). 2021 Nov 5;13(21):5551. doi: 10.3390/cancers13215551 (PMC8583077; doi:10.3390/cancers13215551)
Supplement: Supplementary file 1 [file cancers-13-05551-s001.zip › supplementary/Supp Fig.pdf]

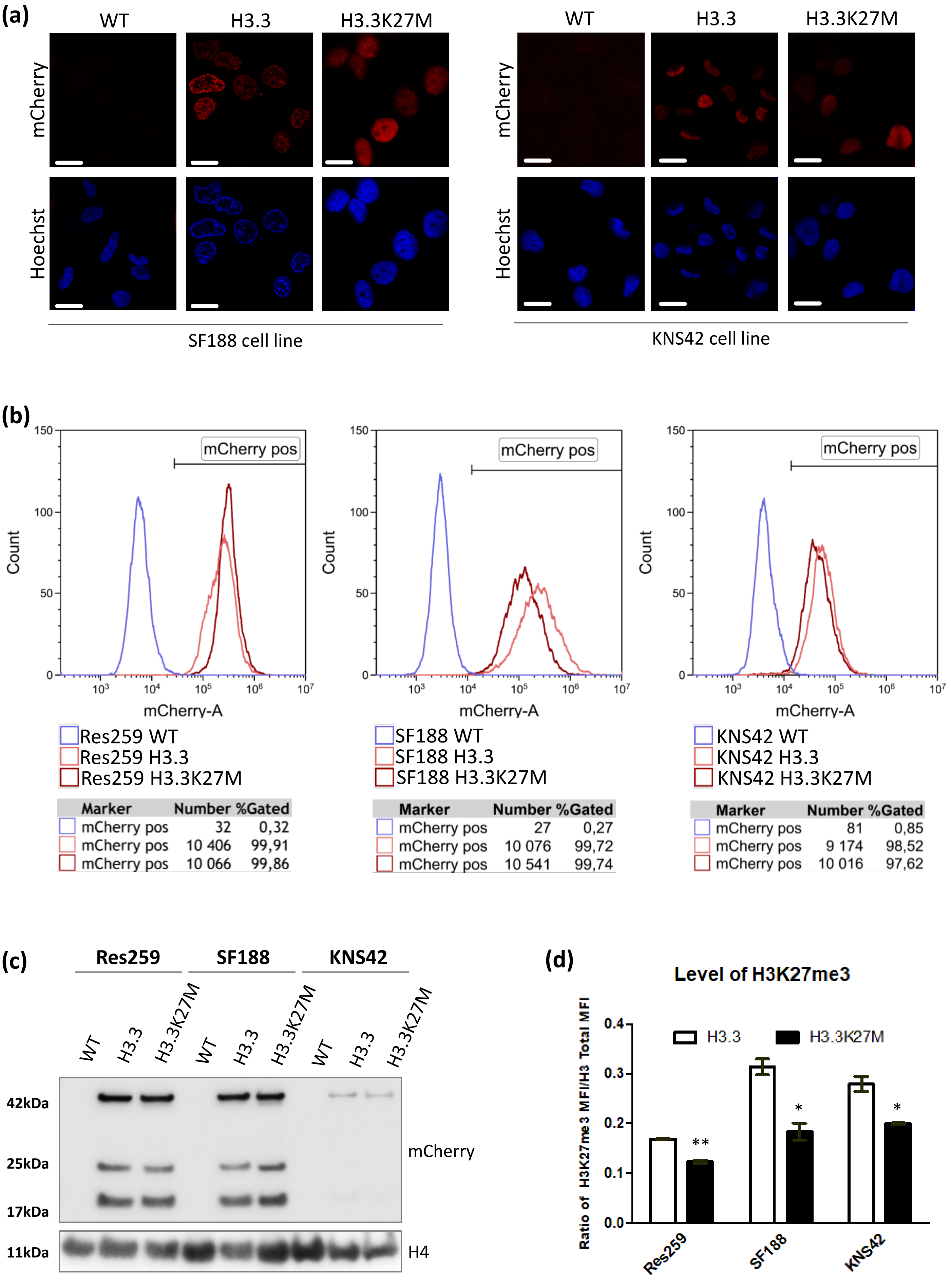

Figure S1: Validation of the H3.3K27M induction models. **(a)** Microscopy pictures of the SF188 and KNS42 cell lines. Untransfected cells (WT) or cells expressing the *H3F3A* mutated (H3.3K27M) or unmutated (H3.3) gene fused to mCherry. Fluorescence was acquired for Hoechst signal (top lane) or mCherry signal (bottom lane). Scale bars 10µm. **(b)** Cell fluorescence was evaluated by flow cytometry based on mCherry signal in Res259, SF188 and KNS42 cell lines. Dark red: H3.3K27M cells. Light red: H3.3 cells. Blue: WT cells. **(c)** Western blot against mCherry was performed in the three cell lines. Histone H4 is used as loading control. **(d)** H3.3K27me3 quantification by Luminex multiplex assay (Active motif service delivery). White and dark histograms represent level of H3K27me3 in H3.3 and H3.3K27M cells respectively.

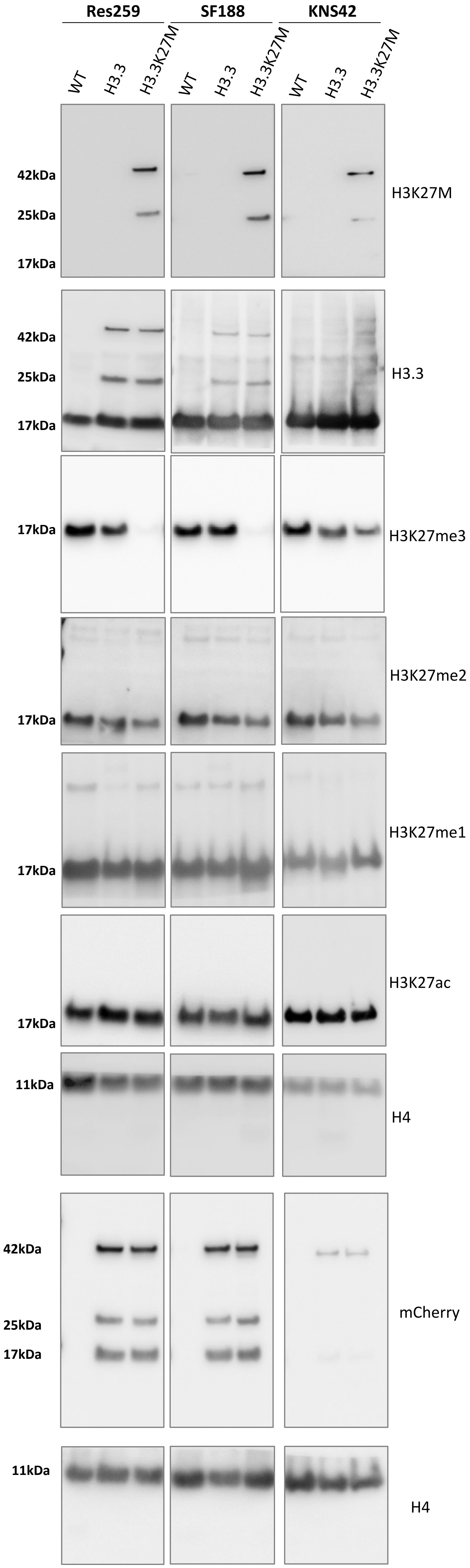

Figure S2: Uncropped Western blot of H3K27M and H3.3 was performed on all cell lines. Other epigenetic marks were also evaluated as H3K27me3/me2/me1 and H3K27ac. H4 was used as a loading control.

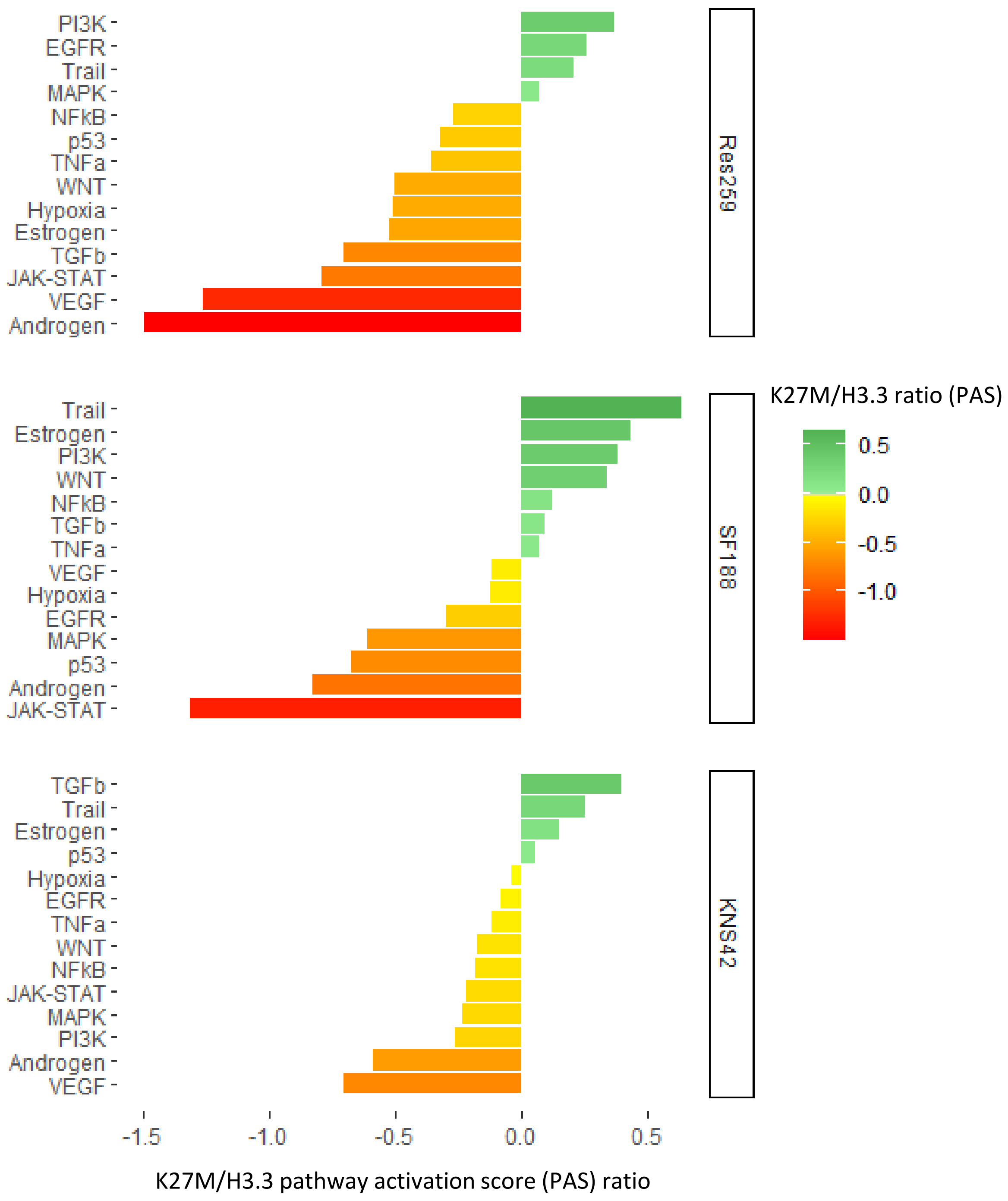

Figure S3. Pathway activation score ratio between K27M and H3.3 conditions in Res259, SF188 and KNS42 glioma cell lines. PROGENy was used to quantify pathway activation per sample based on normalized expression values. For each cell line, we plotted the ratio between pathway activation scores of the K27M and H3.3 conditions. Bars are color-coded with a red (negative K27M/H3.3 ratio) to green (positive K27M/H3.3 ratio) gradient to represent the upper activity of biological pathways in glioma cell lines carrying H3.3K27M mutation.

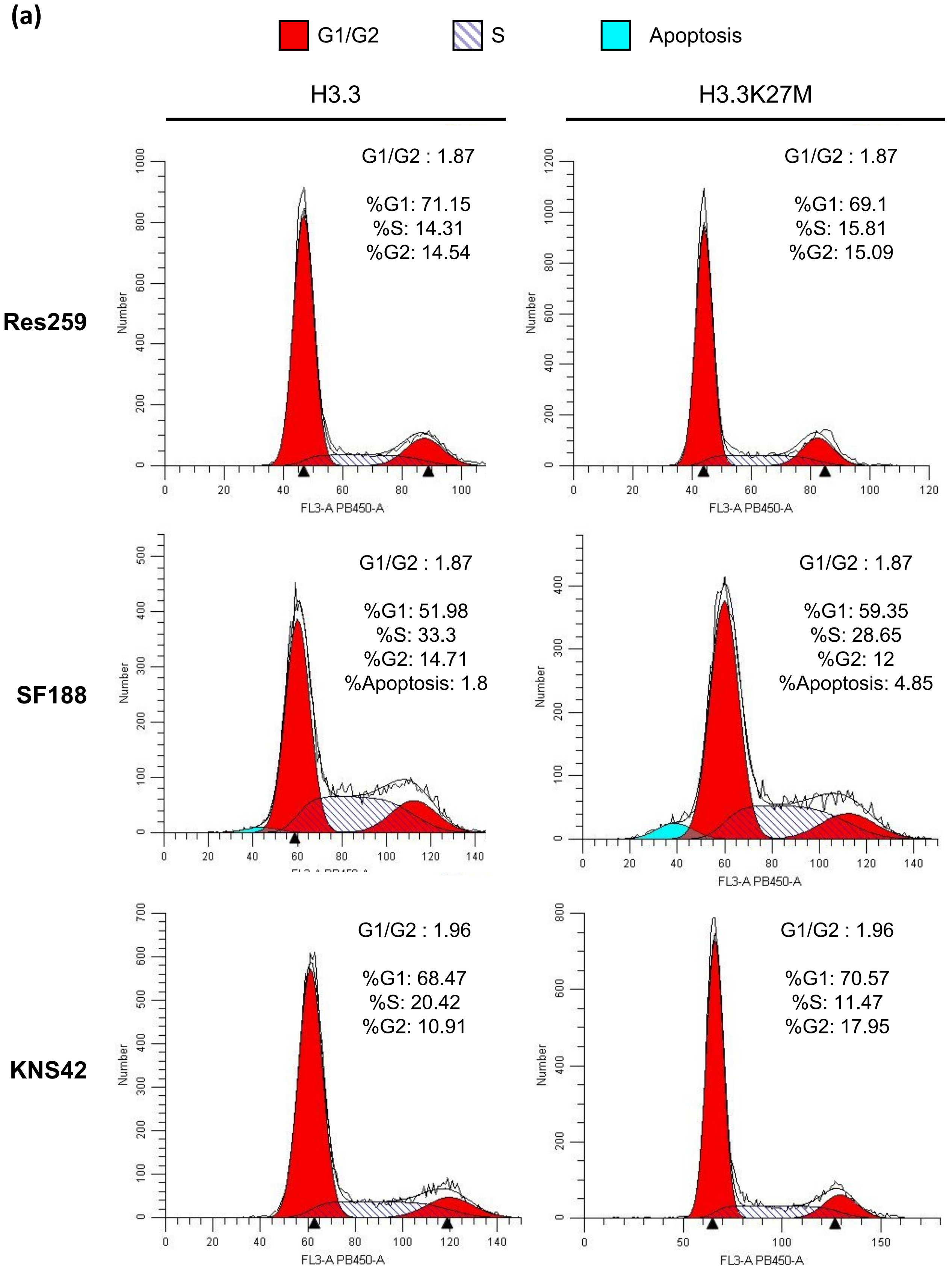

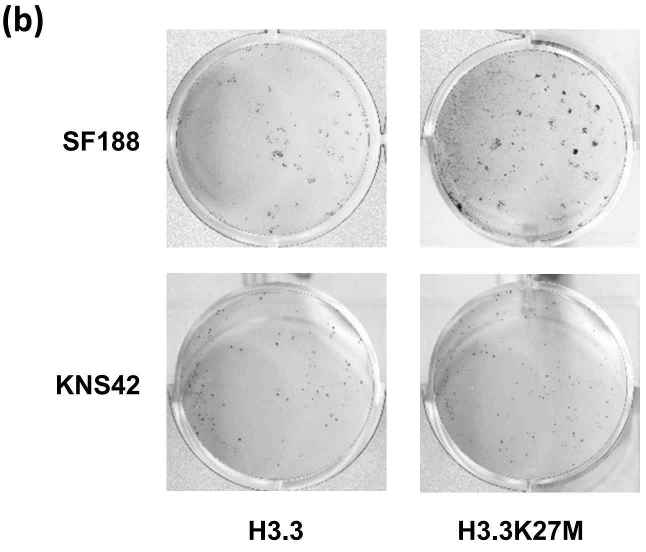

Figure S4: Impact of the H3.3K27M on cell cycle and clonogenic properties. (a) Cell cycle analysis was performed on Res259, SF188 and KNS42 by flow cytometry, with DNA content reflected by the signal of Vybrant™ DyeCycle™ Violet Stain. Analysis was performed on Modfit software. Percentage was calculated setting the ratio G1/G2. **(b)** Pictures of colonies formed by Res259 and KNS42 -H3.3 and -H3.3K27M cells 10 days after seeding.

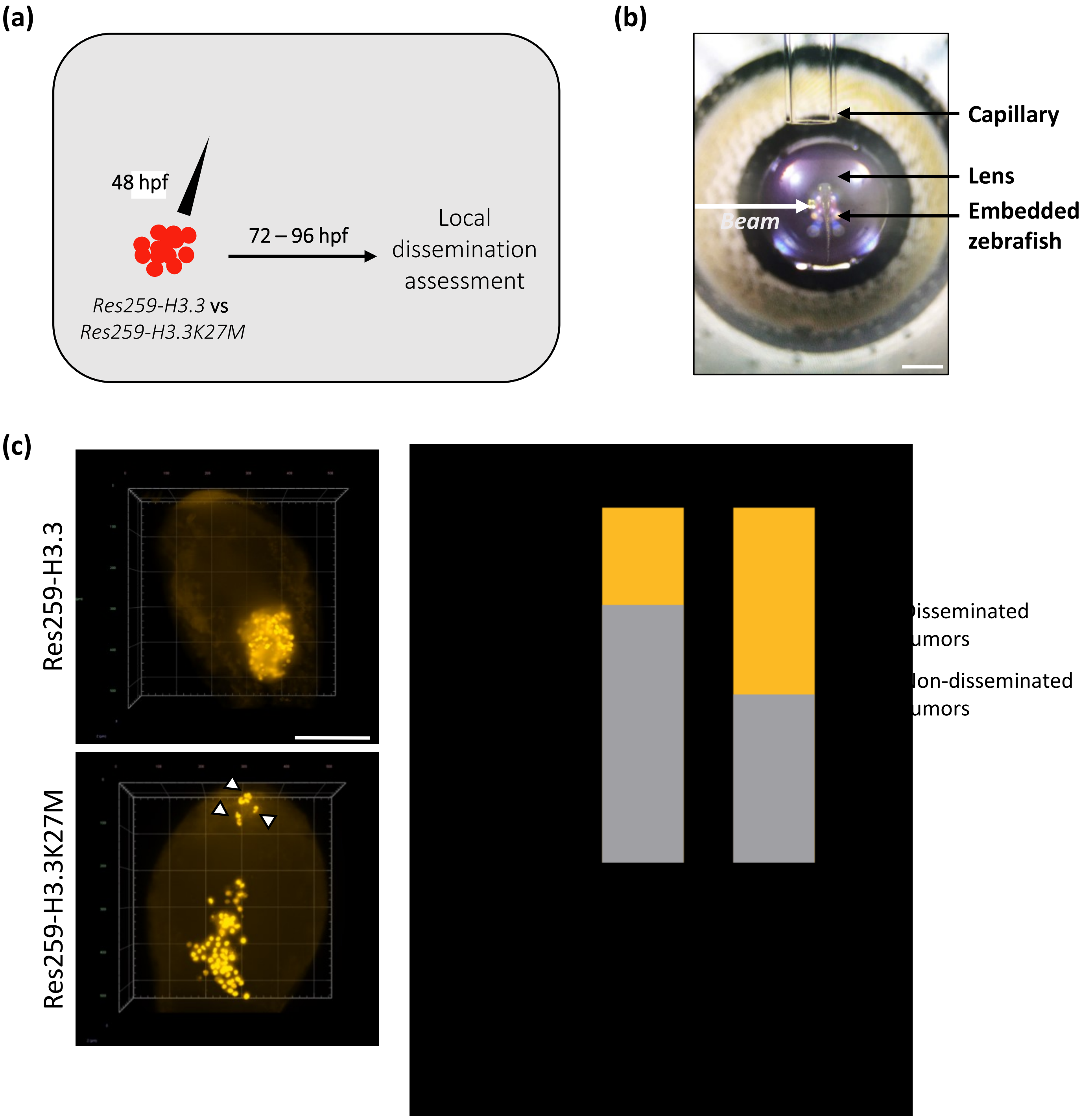

Figure S5: Local dissemination of Res259-H3.3 and -H3.3K27M xenografts in zebrafish embryos. (a) Zebrafish model for the study of local dissemination of xenografts. **(b)** Picture of the set-up for tumor imaging using Selective Plane Illumination Microscopy (SPIM). Scale bar = 2 cm. **(c)** 2D projections of tumors (left panel) and quantification (right panel) of Res259-H3.3 *vs* Res259-H3.3K27M local dissemination in the yolk sac 48 h after tumor grafting. Statistical significance was calculated with the Chi-square ($\chi 2$) test and p-value appears on the corresponding graph. Scale bar = 200 µm.
